# Effect of Citrullus colocynthis hydro-alcoholic extract on hormonal and folliculogenesis process in estradiol valerate-induced PCOs rats model: An experimental study

**Published:** 2017-10

**Authors:** Mohammad Hossein Barzegar, Homayoun Khazali, Seyyed Mehdi Kalantar, Arezoo Khoradmehr

**Affiliations:** 1 *Department of Physiology, Faculty of Biological Sciences, Shahid Beheshti University, Tehran, Iran. *; 2 *Research and Clinical Center for Infertility, Yazd Reproduction Sciences Institute, Shahid Sadoughi University of Medical Sciences, Yazd, Iran.*

**Keywords:** Polycystic ovary syndrome, Gonadotropins hormones, Follicugenesis, Rat Citrullus colocynthis

## Abstract

**Background:**

Citrullus colocynthis (CCT) is used as the anti-diabetic and antioxidant agent. Polycystic ovarian syndrome (PCOS) is a reproductive disorder which level of gonadotropins and sexual hormones are imbalanced.

**Objective:**

We evaluated the effect of CCT hydro-alcoholic extract on hormonal and folliculogenesis process in estradiol valerate-induced PCOs rats’ model.

**Materials and Methods:**

40 female adult Wistar rats divided into five groups (n=8each: Group I (control) only injected by sesame oil as estradiol valerate solvent, group II (Sham) was orally received normal saline after estradiol valerate- induced polycystic ovarian syndrome (4 mg/rat estradiol valerate, intramuscularly), and three experimental groups, that after induction of PCOS within 60 days, received orally 50 mg/kg CCT extract (group III), 50mg/kg metformin (group IV), and CCT extract+ metformin (group V) for 20 days. The serum concentration level of luteinizing, testosterone and follicle stimulating hormones were measured using ELISA method and the serum concentration level of glucose were measured using the oxidative method (glucose meter). Histological study of ovary tissue carried out by hematoxylin-eosin staining.

**Results:**

There was a significant reduction in luteinizing hormone and testosterone in III-V groups compared to Sham group, whereas follicle stimulating hormone in III-V groups was not significantly changed in comparison with Sham group. Histological investigations showed a significant increase in number of preantral and antral follicles and corpus luteum in the experimental groups compared to group II.

**Conclusion:**

Marked improvement in hormonal and histological symptoms of PCOS may be due to CCT effects hence, CCT can potentially be considered as an effective drug for treatment of PCOS.

## Introduction

Polycystic ovary syndrome (PCOS) is the most common endocrinopathy of women, with a prevalence of 6.5-6.7% among premenopausal women (1, 2). The PCOS is also associated with obesity, insulin resistance, diabetes, hyperinsulinemia Hypertension, and dyslipidemia (3, 4). Munir and co-workers demonstrated that insulin activates ovarian P450c17 mRNA expression and enzyme activity through its receptors in theca cells. This action mediated via phosphoinositide 3-kinase (PI3K)/ protein kinase B (PKB) pathway (5). Baillargeon and colleague showed that excessive insulin synthesis may intensify further androgen synthesis in PCOS patients (6). Weight gain is usually associated with an exacerbation of symptoms, while weight loss usually improves the symptoms and endocrine and metabolic disturbances (7, 8). Many of the PCOS patients were identified by increased serum level of luteinizing hormone (LH) and normal or decreased serum level of follicle stimulating hormone (FSH) (9-11). This is explained by an increased pulse frequency of the hypothalamic gonadotropin-releasing hormone (9). Due to the extra production of androgens from theca cells the theca cell layer may thick in follicles of PCOS (12). In addition, insulin increases the response of the theca cells to LH, resulting in increased androgen production (13, 14), and hyperinsulinemia is common in women with PCOS (3). Classical morphology of PCOS includes ovarian cortical thickening, multiple tiny capsular follicular cysts, hyperplasia, luteinized inner theca, stromal hyperplasia and multiple immature follicles, which is an indication of cessation of follicle genesis. The size of ovary ranges from normal size to a very large size (the volume of the ovary increases to more than 10 cm). In ultrasonography, histologic findings appear as a peripheral ring of at least eight small follicles (610 mm diameter) (15). The pathogenesis of PCOS is multifactorial and far from completely understood. Multiple causative mechanisms are discussed: 1) interaction between certain genes and environmental factors (16, 17), 2) dysfunction/regulation by the gonadotropins and intra ovarian factors, and 3) hyperinsulinemia as well as hyper androgenic. 

The citrullus colocynthis (CCT) belongs to the family of cucurbitaceae and grows in arid climate. This plant, also known as bitter apple and bitter cucumber, is famous for its medicinal value throughout the world and in particular in Asia and Africa (18). CCT contains active substances such as poly phenolic components, which are used as anti-diabetic, anti-hypertensive immune-stimulant and antioxidant agents (19-21). Benariba and co-workers assayed the anti-oxidative effects of CCT seeds extract at a concentration of 2000µg/ml in a 1-1 diphenyl-2-picrylhydrazyl. They reported that flavonoids, isosaponarin, isovitexin and isoorientin 3’-O-methyl ether, isolated from the fruits of CCT have significant antioxidant properties (22). Barth *et al* showed that CCT extract was found free of toxic effect on liver at concentration up to 100µg/ml/kg body weight, but higher concentrations seem to have some degree of toxic effect on liver (23). Marzouk and colleagues evaluated the effects of immature fruits and seeds organic extracts of CCT on inflammatory and analgesic process. All results showed an important analgesic and anti-inflammatory activities at different doses (0.5 and 1 mg/kg for anti-inflammatory and 0.05 and 1 mg/kg for analgesic effect) without inducing any side effects (24). Huseini *et al* studied antidiabetic effect of fruit powder of CCT on 50 patients with type II diabetes mellitus. Patients were divided into two groups (n=25). Each group was administrated three times fruit powder of CCT capsules and placebos every day for 60 days. The results obtained from fasting blood sugar, glycosylated hemoglobin, triglycerides, total cholesterol, LDL, HDL, urea, creatinine, alkaline phosphatase, alanine transaminase and aspartate transaminase measurement, elucidated a significant decrease in serum sugar and glycated hemoglobinin in treated group with CCT, however no toxic effect was observed in liver and gastrointestinal system in both groups (25). Sherma et al showed that male rats treated with methanolic extract of CCT exhibited significant (p≤0.01 and p≤0.001) reduction in the level of serum testosterone, FSH and LH in comparison whit controls (26). 

Here, our main goal was to evaluate the effect of 50 mg/kg/day CCT extract and metformin in PCOS rats compared to control group on the mean plasma concentrations of FSH, LH, testosterone, and glucose. Also the numbers of primordial, primary, preantral and antral follicles and. corpus luteum were evaluated during folliculogenesis process.

## Materials and methods

**Plant materials and extraction**

Fresh plants in large quantities were collected from the city of Ardakan in the province of Yazd-Iran in summer. The plant was identified by Department of Biology, Yazd University, and a voucher specimen (C.C-01.01) deposited in the herbarium of the faculty of the biology of Yazd University-Iran. Fruits were thoroughly washed using deionized water, and mopped with tissue paper and air-dried in a shade to prevent the decomposition of chemical constituents. All seeds were manually separated from the pulp of the fruits. The dried pulp of fruits was homogenized with a grinder (Muleinex) to fine powder before extraction. The pulp powder from individual CCT (250 gr) was extracted with 100 ml of water/ethanol mixture (20/80 ml) for 24 hr. Ethanol-soluble portions were pooled from the 300 ml filtrate. The pooled hydro-ethanolic extracts were concentrated by rotary evaporator at 45^o^C (24). The crude extracts (10 gr) was dissolved in freshly prepared normal saline (0.9%) to a final stock solution (10 mg/ml), which was used later to administer 150 μl (50 mg/kg) of the extract to the treatment group.

**Animals**

Female adult Wistar rats weighing 190±20 gr were obtained from Yazd Reproductive Sciences Institute, Yazd, Iran. They were kept in polypropylene cages and left for 2 wk for acclimatization to animal room maintained under controlled conditions (12 hr light/dark cycles at 22±2°C) on standard pellet diet and water ad libitum. Animals were divided into five groups (I-V) (n=8/each group). 

**Experimental design and sample preparation**

Group I (control) only injected by sesame oil as estradiol valerate solvent. In order to PCOS inducing single dose of estradiol valerate (Aburaihan Comoany-Iran, 2ml/day) was injected intramuscularly for each rat in other groups (II-V). To ensure inducing PCOS, from the 10th to the 60th day of injection vaginal smears was prepared daily and stained with Giemsa and examined specimens by light microscope (Olympus CX21, Japan). After 10 days, rats whose estrous cycle was irregular and stopped in metestrus or diestrus was applied as PCOS model (27).

Group II (sham) only received daily 1ml normal saline 0.9% orally. Three experimental groups (III-V) received orally 50 mg/kg/daily CCT extract (group III), 50mg/daily metformin (group IV) and in order to investigate the possibility of co-operation and exacerbation of the therapeutic effect of the extract and metformin, the group V received orally combination of 50 mg of CCT extract (50mg) +metformin (50 mg) for 20 days. This treatment carried out for three weeks for group V. Administrated doses of CCT extract and metformin were in line with the previous study(28). At the end of the third week, under anesthesia, blood sample collected by heart for hormone analysis and ovaries were removed for tissue processing and hematoxylin and eosin method for histology and morphometry. Serum level of glucose was assayed by glucose meter (San Diego CA 92121.USA). The serum levels of LH, FSH, and testosterone were evaluated by Rat/mouse ELISA Kit (Cosmo Bio Co. Ltd. Japan).These measurements were made according to manufacturer's kit. We dispensed 50µl of standards, sample, and control solution into wells. This was followed by adding 100µl of enzyme conjugate, tetramethylbenzidine and stop solution into each well and optical density was read at 450 nm with ELISA reader (VersaMax Elisa. Molecular Devices USA). 

Ovaries were fixed in bouin fixative for 24 hr. Specimens were dehydrated, embedded in paraffin wax and serially sectioned at 6µm thickness on rotary microtome (Didsabz- Iran). This was followed by clearing, hydration and hematoxylin and eosin staining. Slides were studied by light microscope (Olympus CX21, Japan). The number of corpus luteum, primordial (an oocyte surrounded by a single layer of flattened granulosa cells), primary (single layer of cuboidal granulosa cells), preantral (more than one layer of cuboidal granulosa cells) and antral follicles were counted according to the following equation:

$\mathrm{N v}=\frac{\sum Q}{\left(\frac{a}{f}\right)h}$

Nv=the number of follicles in volume unit of the ovary, ∑Q= number of follicles counted in 10 sections from each specimen, h" is the distance between selected sections and a/f was calculated from area/field (29). In this study from ten slides, one was used for counting and thickness of slides was 6µm. 

**Ethical consideration**

All procedures performed in studies involving animal experimental methods were in accordance with the Helsinki declaration and Ethics Committee of the Research and Clinical Center for Infertility, Shahid Sadoughi University of Medical Sciences, Yazd, Iran approved the study protocol (IR-SSU-RSI-REC-1394-1).

**Statistical analysis**

Statistics were performed using SPSS software (statistical package for the social sciences, version 17.0, SPSS inc, Chicago, Illinois, USA). The data were expressed as mean±SD. ANOVA test was used for comparison of the data between different groups. Analyses were considered as statistically significant at p<0.05 and p≤0.001.

## Results

**Figure 1:**
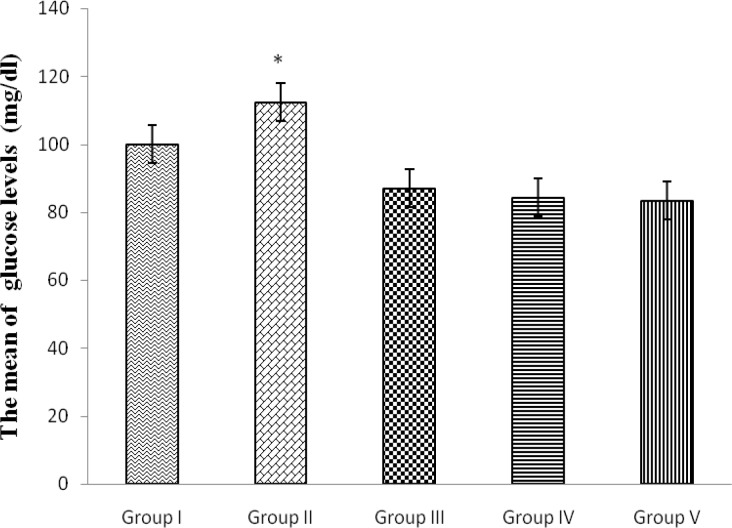
The mean serum levels of glucose (mg/dl) in control (group I), induced PCOS or sham (group II), Citrullus colocynthis extract (group III), metformin (group IV) and Citrullus colocynthis extract and metformin (group V) (n=8/each). Data were analyses by one-way ANOVA test.* shows significant difference of sham group with III, IV and V groups (p≤0.001

**Figure 2:**
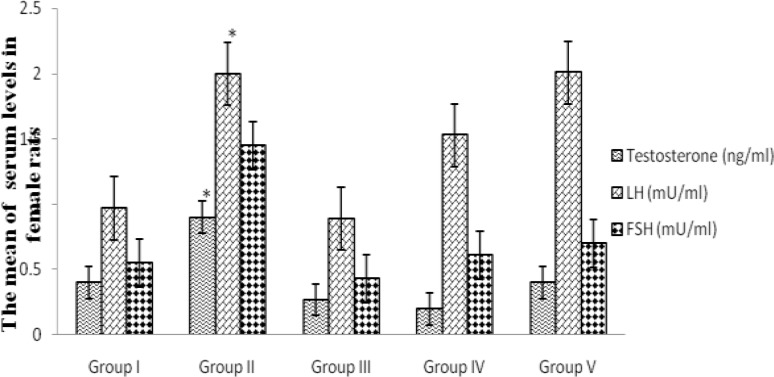
The mean serum levels of Testosterone (ng/ml), LH (mU/ml) and FSH (mU/ml) in control (group I), induced PCOS or sham (group II), Citrullus colocynthis extract (group III), metformin (group IV) and Citrullus colocynthis extract and metformin (group V) (n=8/each). Data were analyses by one-way ANOVA test.* shows significant difference of sham group with III, IV and V groups (Testosterone, LH, and FSH as the level of p≤0.001, p≤0.001 and p=0.063, respectively

The results of this study demonstrated that glucose concentration in serum of PCOS rats was significantly increased compared to controls (115±9.54 and 94.66±10.32 mg/dl respectively) p≤0.000, however, the glucose concentration in the experimental groups was similar (group III 84.33±5.60 mg/dl, group IV: 87±5.29 mg/dl, group V: 83.33±4.84 mg/dl) (Figure 1). The mean serum level of testosterone (0.90±.05 ng/ml) and LH (2.00±0.05 ng/ml) significantly increased in group II compared to the control group (0.40±0.03 ng/ml, p≤0.001 and 0.97±0.08 ng/ml, p≤0.001 testosterone and LH respectively) (Figure 1). Results from the present study show the mean serum level of FSH had no significant variations between any groups 0.55±.03 (control), 0.45±.04 (group II), 0.61±.03 (group III), 0.43±.05 (group IV), 0.70±.05 mU/ml (group V) (p=0.063) (Figure 2).

**Figure 3:**
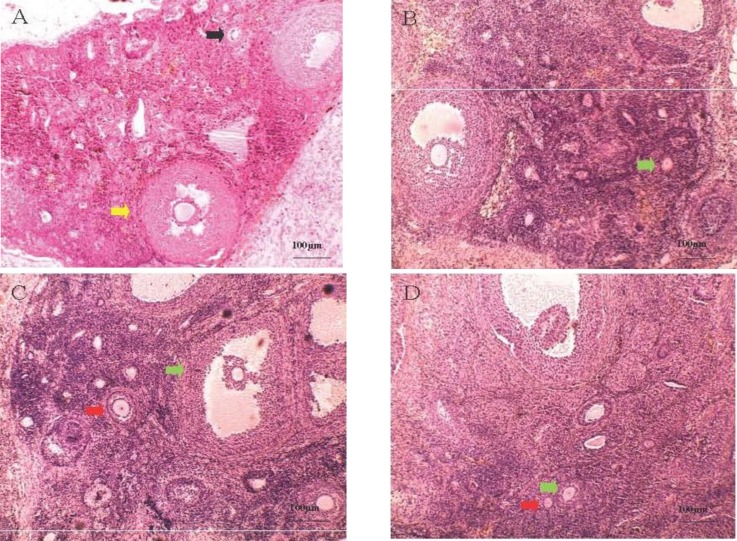
a) Group II: yellow and black arrows show the development of preantral and primordial follicles respectively. b) Group IV: green arrow shows the development of primary follicles. c) Group III: green and red arrows show the development of antral and primary follicles respectively. d) Group V: green and red arrows show the development of primary and primordial follicles respectively. Animals were divided into control (group I), induced PCOS or sham (group II), plant extract (group III), metformin (group IV) and plant extract and metformin (group V) (n=8/ each group) (magnification 200X

**Figure 4:**
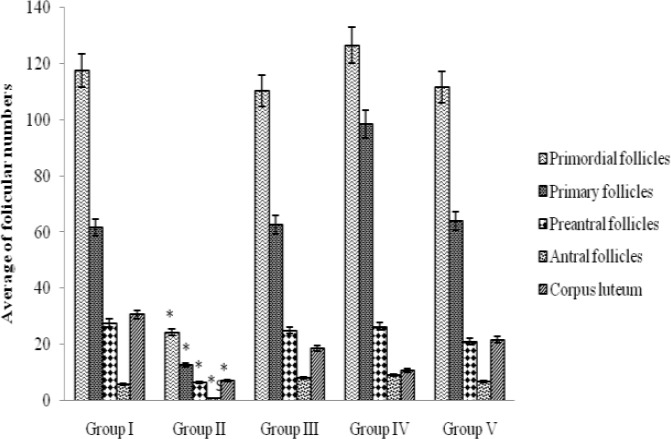
Average number of primordial, primary, parenteral and antral follicles and corpus luteum in female rats

Microscopic images of ovary sections showed that the development of follicles process was attenuated in group II (Figure 3). The number of primordial follicles in I, II, III, IV, and V groups was 117.66±2.51, 24.33±4.04, 110.33±27.22, 126.66±14.46 and 111.66±25.54 in volume unit of the ovary (Nv), respectively (Figure 4). These data showed a sharp decrease in the number of primordial follicles in group II compared to other groups (p=0.002). The number of antral follicles in group II was one sixth preantral follicles (p=0.05 and p=0.001, respectively), while the proportion in other groups was not more than a quarter (The number of preantral follicles in I, II, III, IV, and V groups was 27.66±2.51, 6.33±1.52, 25±3.60, 26.33±4.61, and 21±6.08, respectively while the number of antral follicles in these groups was 6.8±2.94, 0.93±0.25, 8±1, 9.06±0.91 and 6.66±1.52 respectively (Figure 4).

## Discussion

The results of this study showed that the serum levels of LH, glucose, and testosterone in treated groups were significantly decreased with respect to group II whereas the serum level of FSH were not significantly changed. Several factors including androgens overproduction with ovaries and adrenal origin, insulin resistance, obesity, gonadotropin dysregulation and heritance fields are involved in PCOS pathogenesis (16, 30). Therefore, probably mechanism of action of CCT can be explained either by the anti-androgenic and anti-diabetic or the ability of extract to reduction resistance to insulin. These results are consistent with the studies that demonstrated CCT extract have anti-androgenic, anti-hyper- glycaemia, anti-oxidative and anti-inflammatory activities. These results are also in agreement with the previous studies that suggested CCT includes diverse antioxidants and anti-inflammatory factors such as flavonoids. Delazar et al evaluated phytochemical constituents of fruit pulps of CCT. They identified that CCT contains anti-inflammatory and anti-oxidative components such as three flavone glucosides, isosaponin, isovitexin and isoorientin 3’-O-methyl ether (31). Giwa *et al* showed that seed oil of CCT, as well as fruit pulps, contain different antioxidants such as diverse flavonoids Luteolin, Quercetin, and Myricetin (32). Zhenzehi *et al* showed that quercetin significantly reduces the plasma level of TNF-a, IL-1, IL-6, and insulin. They also demonstrated that this flavonoid decreases the granulosa cell nuclear translocation of NF-κB in the insulin-resistant PCOS rat model. They proved that qurcetin inhibits toll-like receptor/NF-kB signaling pathway in ovarian tissue of the PCOS rat (33). Shah and co-workers treated PCOS rats with metformin and quercetin orally at dose 150mg/kg after six months. They founded that both treatment improved luteinizing hormone, testosterone, and insulin. They also demonstrated that quercetin inhibits Phosphatidylinositol-4, 5-bisphosphate 3-kinase (PI3K) activity and decreases the expression of an ovary determining gene of CYP17A1 (34). Sivalingam et al showed that metformin, as an anti-hyperglycaemia can,is used to promote ovulation in PCOS rats. This medicine interacts with PI3K/ACT/mTOR signal pathway (35).

Ovarian hyper androgenism is mainly related to an inherent steroidogenic defect of theca cells in PCOS. The level of LH and insulin plasma levels appear to enhance innate abnormality of theca steroids synthesis (36). Additionally, Increasing the LH pulse frequency and amplitude leads to perdurable increased LH levels. This factor may directly enhance theca androgen synthesis. However, it has been suggested that high levels of LH could be the result of impaired negative feedback on LH secretion, which is, caused by excessive androgen action on the hypothalamic-pituitary axis (37). Insulin seems to be a starting factor that intensifies the inherent dysregulation of theca steroids synthesis in PCOS. It seems that the effect of insulin to stimulate androgen synthesis is independent of LH in PCOS ovarian theca cells (38). By keeping the plasma glucose level in the normal range, it was shown that reducing the insulin level by diazixide results in decreasing the androgen synthesis in lean women with typically PCOS and normal insulin sensitivity. These findings showed that androgen synthesis pathway is sensitive to insulin (6).

Other studies have suggested that chronic low-grade inflammation is due to disruption balance between pro-inflammatory and anti-inflammatory cytokines in PCOS (39). The increased tumor necrosis factor-alpha (TNF-α) production by mononuclear cells, in response to hyperglycemia, may further intensify metabolic and hormonal abnormalities in PCOS. TNF-α via its own receptor and via activated nuclear factor kappa-light-chain-enhancer of activated B cells (NF-kB (induces serine phosphorylation of insulin receptor substrate type 1 (IRS1), Destructive insulin resistance (40). Investigation of human obesity and insulin resistance has demonstrated a clear relation between the chronic activation of pro-inflammatory signaling pathways and decreased insulin sensitivity. For example, high levels of TNF-α, interleukin-6, and interleukin-8 have all been reported in various diabetic and insulin-resistant state (41).

The roles of kinases in the initiation of inflammation and the immune response are linked to the translocation of the transcription factor NF-kB to the nucleus. Cytoplasmic levels of NF-kB levels are regulated in part by its binding to the inhibitor of kappa B molecule (IkB (which is phosphorylated via an IkB kinase and degraded in during cell activation. The actions of IkB kinase are therefore linked to the translocation of NF-kB to the nucleus and subsequent gene expression. According to the previous studies where it was shown that apigenin inhibites activity of IKB kinase in mouse macrophage, can be concluded that the inhibition of this key regulatory enzyme is critical in control of pro-inflammatory (42).

The results of our study show that in treated groups with CCT extract (group III) for 20 days, the plasma level of LH and testosterone decreased while plasma level of FSH did not change significantly compare to the group II. These results can be related to anti-inflammatory and anti-oxidative effect of CCT flavonoids. Flavonoids can act as antioxidants to prevent diseases by modulating oxidative stresses in the body (43). Therefore, the discovery of plant compounds with anti-inflammatory effect such as flavonoids can lead to the development of plant drugs for the treatment of many pro-inflammatory diseases. Since the variety of immune response and pro-inflammatory process mediated by cytokines such as TNF-α and interleukin-6 can be a useful strategy for the treatment of inflammatory disorders. This pathway represents an important and attractive clinical target for compounds that selectively interfere with the inhibition of necrosis factor kappa B (44).

The results of this study showed that in the group II compared with the other groups significantly decreased the number of follicles. These results are consistent with previous studies (45). According to the studies, the cause of the failure can be an excessive expression of LH receptors in the cells of preantral follicles attributed granulosa cells (46, 47). Respond more LH causes the cells to luteinizing too early and as a result, do not continue their growth process (48). Also, Loumaye and co-workers showed that stopping folliculogenesis process can be caused by elevated ovarian androgen levels that will be established under the influence of LH (49). Jonard and co-workers showed that high concentrations of plasma insulin can also cause overexpression of the receptor gene of LH in granulosa cells and therefore luteinizing occurs earlier than normal preantral follicles (45). These evidence show that improvement of folliculogenesis in the treated groups with CCT extract is due to the ability of flavonoids to decrease serum level of LH, testosterone, and insulin.

## Conclusion

Our study showed that CCT extract can improve hormonal and follicle development disorders in PCOS rats. This plant also can decrease the serum level of LH and testosterone and glucose. Probably these effects can be related to flavonoids of CCT.
